# Beyond Atrial Fibrillation: A Case of Thyrotoxicosis-Associated Ischemic Stroke in a Young Patient

**DOI:** 10.7759/cureus.102759

**Published:** 2026-01-31

**Authors:** Afnan Tayeb, Selma Later, Mohamed Nassef

**Affiliations:** 1 Internal Medicine, Al Qassimi Hospital, Sharjah, ARE; 2 Anesthesia and Critical Care, Al Qassimi Hospital, Sharjah, ARE; 3 Critical Care Medicine, Al Qassimi Hospital, Sharjah, ARE

**Keywords:** atrial fibrillation, cryptogenic stroke, hypercoagulability, hyperthyroidism, moyamoya disease, stroke in young adults, thyrotoxicosis

## Abstract

While it is presumed that thyrotoxicosis is a contributing factor for cardioembolic stroke secondary to atrial fibrillation (AF), cerebrovascular events in patients without cardiac arrhythmias remain exceptionally rare. We present the case of a 41-year-old African female patient with no traditional stroke risk factors who presented with sudden-onset left hemiplegia and dizziness (National Institutes of Health Stroke Scale (NIHSS) 9). Imaging revealed a right middle cerebral artery (MCA) territory infarct with hemorrhagic transformation and malignant edema (midline shift 3 mm), necessitating emergent decompressive craniectomy. Postoperatively, she developed refractory fever, extreme tachycardia, and hypertension despite broad-spectrum antibiotics for presumed sepsis. Endocrine workup confirmed thyroid storm, and the patient was treated aggressively with propylthiouracil, cholestyramine, and bisoprolol in addition to stress-dose glucocorticoids. Subsequent labs uncovered refractory hypercalcemia, prompting further evaluation that confirmed hyperparathyroidism. Notably, her presentation occurred in the absence of AF. The cardiovascular effects of hyperthyroidism are well-established, with sinus tachycardia and AF being the most common electrocardiographic abnormalities. Emerging evidence further implicates thyroid dysfunction in coagulation pathway dysregulation, which may predispose patients to thromboembolic events independent of cardiac arrhythmias. This case underscores the importance of considering thyrotoxicosis in cryptogenic stroke, even without AF, and highlights gaps in current guidelines regarding anticoagulation therapy for this high-risk population.

## Introduction

Thyroid storm is a life-threatening complication of hyperthyroidism, often characterized by high fever, fast heart rate, and altered mental status [1]. It is thought that excess thyroid hormones are linked to increasing the risk of stroke through atrial fibrillation (AF) [2].

However, strokes occurring independent from abnormal heart rhythms in the presence of thyroid storm remain poorly understood. Only isolated case reports describe stroke in thyroid storm without AF [3]. Growing evidence suggests that thyroid hormone itself may promote abnormal blood clotting, increasing stroke risk even in the absence of arrhythmia [4], though this remains underrecognized.

We report a case of severe middle cerebral artery (MCA) infarction in the setting of thyroid storm without AF, complicated by hyperparathyroidism-related hypercalcemia. This case highlights the need to consider thyroid disease as a potential cause of unexplained strokes and emphasizes ongoing therapeutic challenges, particularly regarding anticoagulation in the absence of clear guidelines.

## Case presentation

**Figure 1:**
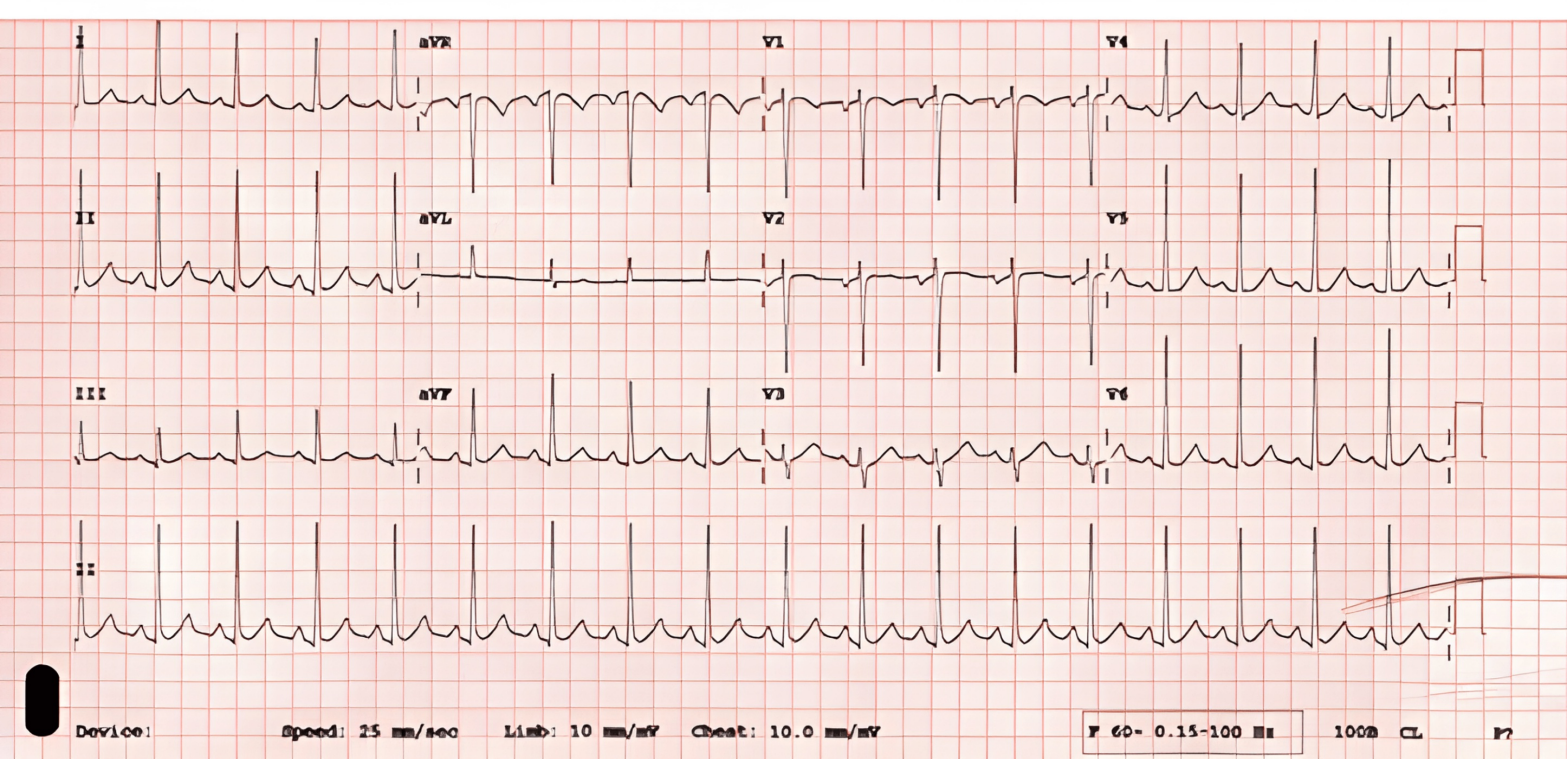
ECG on admission demonstrating normal sinus rhythm

A 41-year-old female patient with no significant past medical history presented with sudden-onset left hemiplegia, right-sided headache, and dizziness after witnessed collapse. On arrival, the patient was found to be hypertensive (146/78 mmHg) and tachycardic (112 beats per minute (bpm)). Physical examination revealed a conscious and alert patient with notable neurological findings of left-sided hemiplegia and dizziness (National Institutes of Health Stroke Scale (NIHSS) score 9); cardiopulmonary auscultation was clear. Electrocardiogram (ECG) revealed sinus tachycardia (Figure 1).

**Figure 2:**
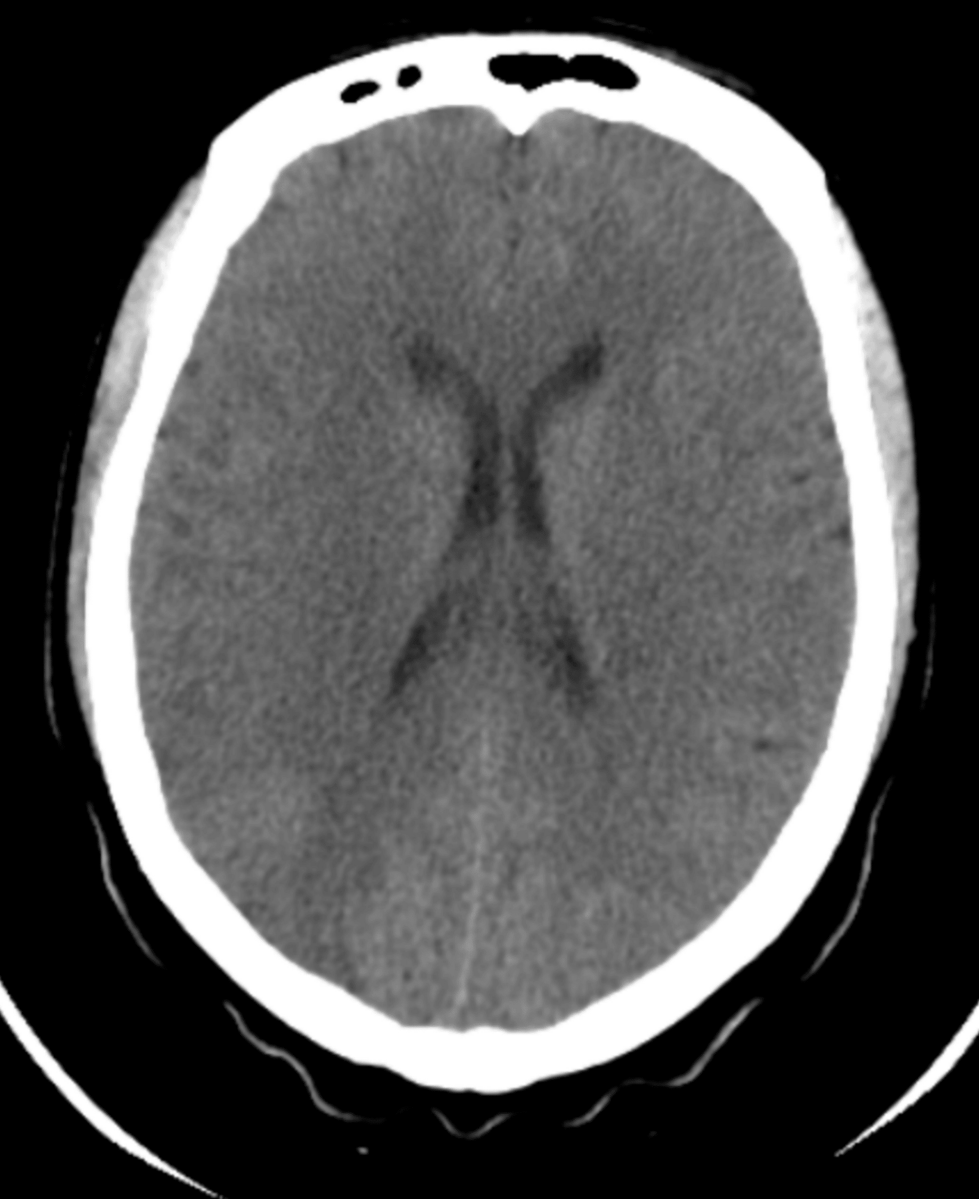
Initial non-contrast CT brain. Normal study.

**Figure 3:**
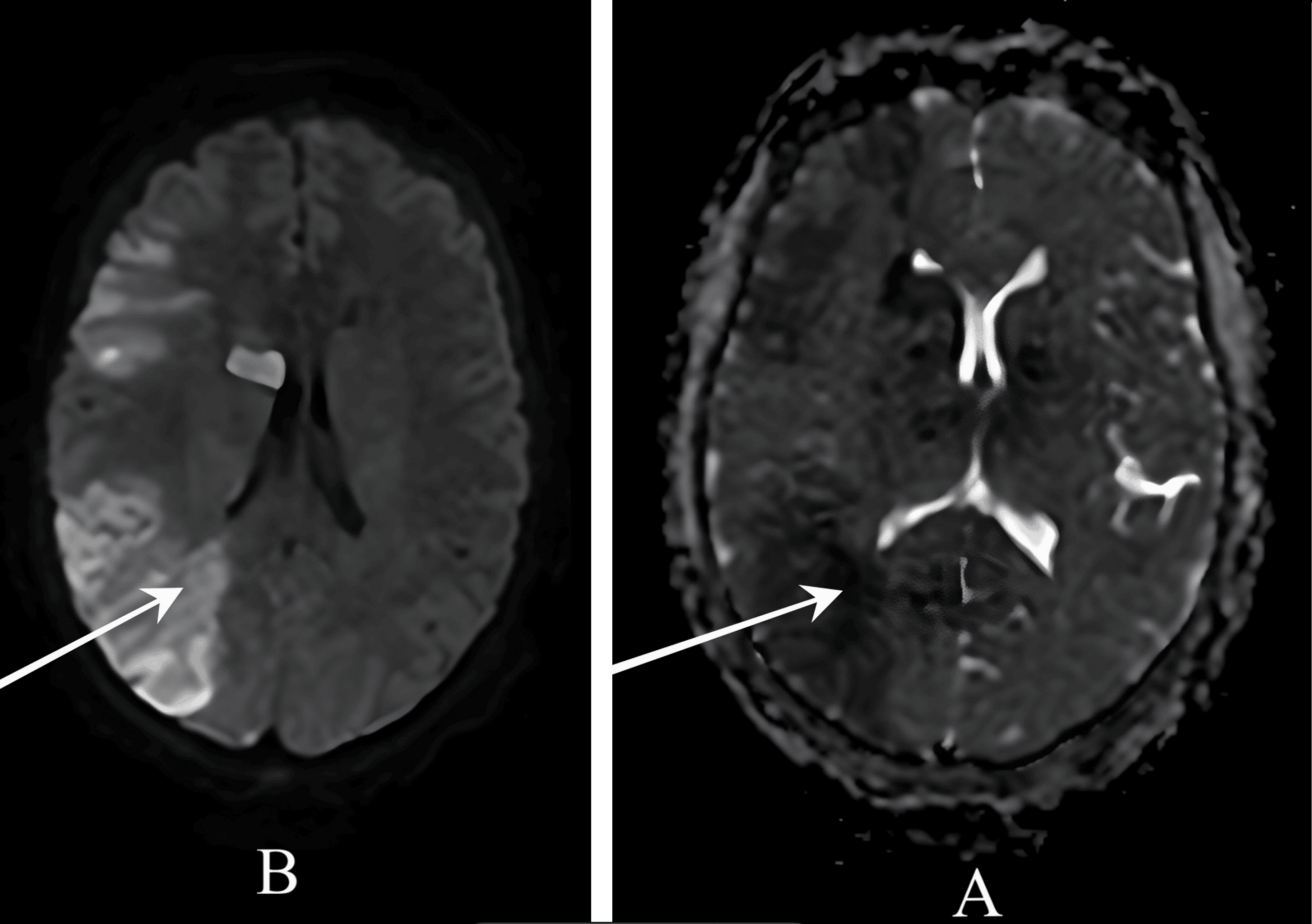
CT Brain demonstrating acute ischemic stroke in the right MCA territory (A) ADC hypo-intensity confirming true diffusion restriction; (B) DWI showing area of restricted diffusion (bright signal) in right MCA territory. MCA: middle cerebral artery; DWI: diffusion-weighted imaging; ADC: apparent diffusion coefficient

Computerized tomography (CT) brain without contrast done initially revealed no signs of acute ischemia or hemorrhage (Figure 2). Due to the high clinical suspicion, magnetic resonance imaging (MRI) of the brain was performed, which demonstrated an acute right MCA territory infarction involving the right parieto-temporal cortex and basal ganglia, with hemorrhagic transformation in the right parietal region (Figure 3).

**Figure 4:**
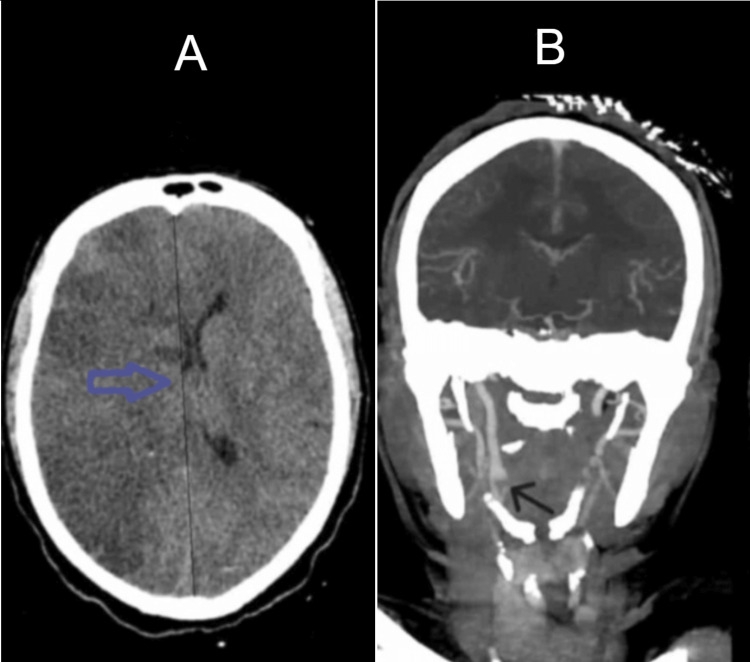
(A) Non-contrast CT head showing malignant infarction of the right MCA territory with ~3 mm midline shift (blue arrow). (B) CT angiography demonstrating thrombus in the right internal carotid artery (black arrow). MCA: middle cerebral artery

Subsequent CT head showed malignant infarction with 3 mm midline shift and partially effaced basal cisterns (Figure 4A). Vascular imaging confirmed right internal carotid artery (ICA) thrombus (Figure 4B).

The patient’s condition deteriorated, with a declining Glasgow Coma Scale (GCS), prompting a right decompressive craniectomy. Intraoperatively, the brain was initially non-pulsatile but improved after decompression. 

Following decompressive craniectomy, the patient was kept sedated and ventilated for 48 hours, with dehydrating measures initiated. Her GCS remained stable at 7/15 (pupils equal and reactive). On postoperative day 2, she developed persistent fever spikes (tympanic temperature 40.5°C) and refractory sinus tachycardia (heart rate >150 bpm) accompanied by elevated blood pressure (averaging 160/70 mmHg).

Initial sepsis evaluation revealed moderately elevated inflammatory markers (C-reactive protein 20-100 mg/L; reference range, 0-3 mg/L, and procalcitonin 0.26-0.9 ng/mL; reference range, 0-0.1 ng/mL), prompting empiric broad-spectrum antibiotics (cefepime and teicoplanin). While sputum cultures later identified *Staphylococcus aureus*, the patient's minimal clinical improvement and disproportionate adrenergic features prompted expansion of the differential diagnosis. A summary of the relevant laboratory results is shown in Table 1.

**Table 1:** Summary of laboratory results CK-MB: Creatine Kinase-Myocardial Band

Laboratory Tests	Patient Values	Reference Ranges
Complete Blood Count		
Hemoglobin	9.0 g/dL	12.0 - 16.0 g/dL
Hematocrit	29.40%	36.0 - 48.0%
White Blood Cell Count	10.05 ×10⁹/L	4.5 - 11.0 ×10⁹/L
Platelet Count	487 ×10⁹/L	150 - 450 ×10⁹/L
Neutrophils	67.20%	40.0 - 70.0%
Lymphocytes	11.20%	20.0 - 45.0%
Basic Metabolic Panel & Renal Function		
Sodium	134 mmol/L	136 - 145 mmol/L
Potassium	4.1 mmol/L	3.5 - 5.1 mmol/L
Chloride	104 mmol/L	98 - 107 mmol/L
Bicarbonate	20.4 mmol/L	22.0 - 29.0 mmol/L
Blood Urea Nitrogen	2.34 mmol/L	2.5 - 7.1 mmol/L
Creatinine	42 μmol/L	53 - 97 μmol/L
Estimated Glomerular Filtration Rate	116 mL/min/1.73m²	≥ 90 mL/min/1.73m²
Uric Acid	323 μmol/L	150 - 360 μmol/L
Glucose	12.9 mmol/L	4.2 - 5.8 mmol/L (Fasting)
Coagulation Profile		
International Normalized Ratio	1.14	0.9 - 1.1
D-Dimer	2.77 mg/L FEU	< 0.50 mg/L FEU
Protein S	49%	55 - 123%
Protein C	86%	70 - 140%
Antithrombin III	95.80%	80 - 120%
Cardiac Enzymes		
Troponin I	< 0.01 ng/mL	< 0.04 ng/mL
CK-MB	0.5 ng/mL	< 3.0 - 5.0 ng/mL
Thyroid Function		
Thyroid-Stimulating Hormone	0.00 mIU/L	0.55 - 4.78 mIU/L
Free Thyroxine	49.1 pmol/L	9 - 20 pmol/L
Free Thyroxine (Follow-up)	21 - 20.88 pmol/L	9 - 20 pmol/L
Thyroid-Stimulating Hormone (Follow-up)	0.01 - 0.02 mIU/L	0.55 - 4.78 mIU/L
Liver Function		
Alanine Aminotransferase	150 U/L	14 - 59 U/L
Aspartate Aminotransferase	163 U/L	15 - 37 U/L
Calcium Metabolism		
Serum Calcium	3.95 mmol/L	2.12 - 2.52 mmol/L
Parathyroid Hormone	15.8 pmol/L	1.59 - 8.49 pmol/L
Phosphate	0.5 - 0.7 mmol/L	0.84 - 1.52 mmol/L
Parathyroid Hormone (Follow-up)	8.2 pmol/L	1.59 - 8.49 pmol/L
Inflammation & Infection		
C-Reactive Protein	20 - 100 mg/L	0 - 3 mg/L
Procalcitonin	0.26 - 0.9 ng/mL	0 - 0.1 ng/mL

The patient was thoroughly investigated for the cause of the infarction. In view of her young age, autoimmune phenomena were suspected, so a complete autoimmune workup was done, including antinuclear antibody (ANA), rheumatoid factor (RF), antineutrophil cytoplasmic antibodies (ANCA), anti-double-stranded DNA (dsDNA), anti-cyclic citrullinated peptide (anti-CCP), anti-extractable nuclear antigen (anti-ENA), including anti-Smith (anti-Sm) antibodies, anti-Ro/SSA, anti-La/SSB, anti-Jo antibodies, anti-ribonucleoprotein antibodies (anti-RNP), lupus anticoagulant, and anti-phospholipid antibodies (APL antibodies), which were all negative.

**Figure 5:**
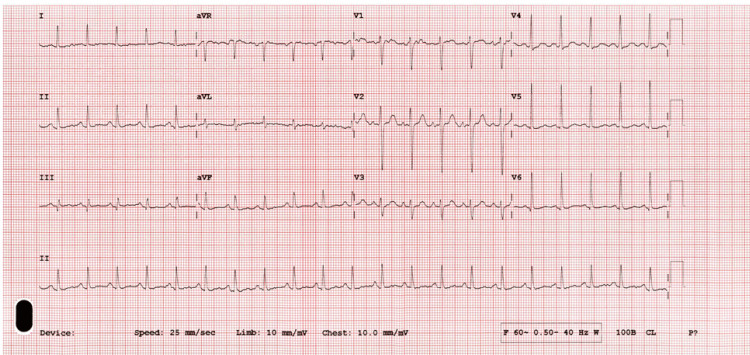
Repeat ECG showing sinus rhythm

**Figure 6:**
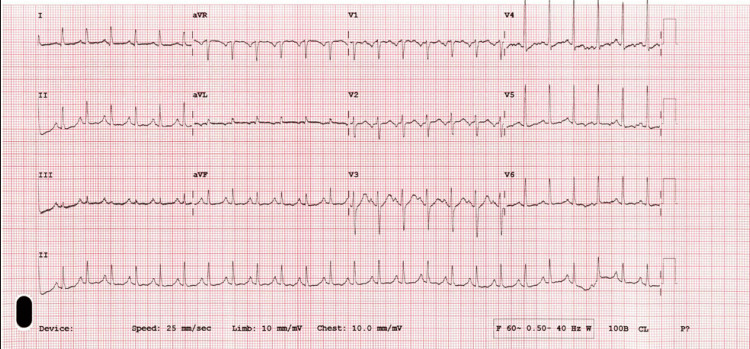
Another repeat ECG showing sinus rhythm

To definitively exclude arrhythmia as a potential underlying etiology, a comprehensive cardiac evaluation was pursued. Transthoracic echocardiography revealed a structurally normal heart without wall motion abnormalities or intracardiac thrombus. Furthermore, serial electrocardiograms were obtained to capture any paroxysmal rhythm disturbances; all tracings consistently demonstrated normal sinus rhythm (Figures 5, 6).

Tumor markers, including alpha-fetoprotein (AFP), carcinoembryonic antigen (CEA), cancer antigen (CA) 19-9, CA 15-3, and CA 125 were negative. Coagulation profile, including Factor V Leiden, anti-thrombin III, protein C, and protein S, came out negative.

However, the evolving clinical picture, particularly the refractory hyperthermia, extreme tachycardia, and persistent hypertension despite sepsis management, raised suspicion for thyroid storm. This was confirmed biochemically with undetectable thyroid-stimulating hormone (TSH) (0.00 mIU/L; reference range, 0.55-4.78 mIU/L) and markedly elevated free thyroxine (fT4) (49.1 pmol/L; reference range, 9-20 pmol/L), diagnostic of a hyperthyroid crisis. Thyroid ultrasound revealed a left lobe nodule within an otherwise normal-sized gland with homogenous texture and unremarkable vascularity.

Treatment was initiated with propylthiouracil (PTU), cholestyramine, and bisoprolol for comprehensive hormone suppression and adrenergic blockade, along with stress-dose glucocorticoids. The absence of alternative stroke mechanisms and the temporal sequence of symptoms strongly suggested that thyrotoxicosis precipitated the cerebrovascular event, with the subsequent physiological stress of surgery exacerbating the thyroid crisis.

Serial thyroid function tests showed fT4 improvement with only mild fT4 elevation (21-20.88 pmol/L) and suppressed TSH (0.01-0.02 mIU/L), allowing PTU reduction and hydrocortisone taper. However, due to elevated liver enzymes: alanine aminotransferase (ALT) 150 U/L (reference range, 14-59 U/L), and aspartate aminotransferase (AST) 163 U/L (reference range, 15-37 U/L), PTU was switched to carbimazole. By one week later, thyroxin level normalized: carbimazole was reduced, cholestyramine discontinued, and beta-blockers continued. Glucocorticoids were maintained at higher doses for concurrent hypercalcemia.

The patient developed moderate hypercalcemia (peak serum calcium 3.95 mmol/L (reference range, 2.12-2.52 mmol/L) accompanied by inappropriately elevated parathyroid hormone (PTH) (15.8 pmol/L, reference range, 1.59-8.49 pmol/L) and persistent hypophosphatemia (0.5-0.7 mmol/L, reference range, 0.84-1.52 mmol/L). The hypercalcemia management included intravenous volume resuscitation, initiation of cinacalcet therapy, and low-calcium hemodialysis as needed, while continuing antithyroid medications and glucocorticoids for the underlying thyroid storm. The calcium levels normalized within 72 hours, with parallel reduction in PTH levels (8.2 pmol/L, normal 1.59-8.49 pmol/L).

The patient remained ventilator-dependent with depressed consciousness post-stroke, requiring a prolonged ICU stay. During this time, she developed recurrent infections (pneumonia, urinary tract infections), which progressed to sepsis and septic shock, along with acute kidney injury, all managed with culture-directed antibiotics, hemodynamic support, and hemodialysis as indicated. Subsequently, she underwent tracheostomy followed by successful percutaneous endoscopic gastrostomy (PEG) tube placement. Her neurological status gradually improved, with regained alertness, spontaneous right-sided movements, and attempts at speech. However, she required ongoing respiratory care and remained dependent on a wheelchair for mobility.

## Discussion

Our case illustrates the rare association between ischemic stroke and thyrotoxicosis. The patient presented with ICA occlusion and MCA infarction and was subsequently diagnosed with thyrotoxicosis. This case emphasizes the importance of considering thyroid disorders as a potential etiological factor of ischemic stroke in the absence of other risk factors in low-risk individuals.

Ischemic stroke is a term that describes the neurological deficits resulting from vasoconstriction/occlusion of brain vessels, leading to decreased blood supply, thus tissue infarction. Well-recognized risk factors include age, hypertension, diabetes, dyslipidemia, as well as modifiable risk factors such as smoking and obesity. It mostly occurs amongst middle-aged or older individuals [5].

Thyrotoxicosis, an endocrine dysfunction, refers to elevated levels of thyroid hormones in the blood, leading to a hypermetabolic state. Neurological disorders related to hyperthyroid include myopathies, hyperkalemic periodic paralysis, and hyperthyroid myasthenia gravis [6]. The known mechanism of cerebral ischemia in hyperthyroidism is the hyperdynamic cardiovascular state, such as tachycardia and arrhythmias, most importantly AF. According to Cai et al.'s case report and literature review on the rare incidence of corpus callosum infarction associated with thyroid storm, the incidence of ischemic stroke associated with a hyperthyroid state is 1%, mostly seen in young adults [6]. Many cases have been identified. A study analysis of two cases by Ivan et al. demonstrated the relationship between thyroid dysfunction and ischemic stroke in the absence of other risk factors [7]. Another case published by Lim et al. about a young woman with recurrent strokes, moyamoya disease, and hyperthyroidism [8].

The pathophysiology of the mechanism of stroke in our patient is yet to be defined. A few possible causes have been identified worldwide. First, it is important to note that paroxysmal cardiac arrhythmias can not be entirely excluded. Second, excess thyroid hormones were found to have contributed to endothelial dysfunction and upregulation of various clotting factors, including factor IX, fibrinogen, anti-thrombin III, von Willebrand Factor (vWF), tissue plasminogen activator inhibitor-1, alongside decreased levels of tissue plasminogen activator (t-PA), leading to thrombotic events according to a study by Erem et al. [9]. Another study in 2006 by Erem found increased levels of factor X in patients with subclinical hyperthyroidism compared to the control group [10]. These findings were supported by a case report by Ren et al. of a patient with Graves' disease and Moyamoya disease who exhibited elevated levels of vWF and coagulation factor VIII, suggesting a hypercoagulable state contributing to cerebrovascular compromise [11]. In our patient, her coagulation profile was within normal except for decreased protein S levels.

Another theory is the autoimmune phenomenon. In our patient, her autoimmune profile was unremarkable. Hsu et al. pointed to the possibility of a cross-reaction between thyroid antibodies and unidentified antigens in the cerebral vessels [12]. Such findings were mostly suggested in patients who had Graves’ disease and moyamoya disease. Leno et al. suggested a similar theory after assessing a case of Down syndrome with moyamoya disease [13]. Additionally, a correlation between antiphospholipid syndrome antibodies and thyroid antibodies has been demonstrated, as antiphospholipid syndrome has been found in patients with Graves’ disease. A control study demonstrated an association between giant cell arteritis and Takayasu arteritis with hyperthyroidism, for which both increase the risk of developing stroke by mediating inflammation and endothelial proliferation [14]. Autoimmunity has been found to be related to atherosclerosis formation due to chronic inflammation and oxidation [15]. Hyperthyroidism was found to increase oxidation and reduce antioxidants in patients with metabolic disorders, contributing to vascular injury [16].

Ku et al. mentioned possible T-cell dysregulation and moyamoya thyrotoxicosis [17], while Zhang et al. concluded an association between thyroid autoantibodies and intracranial stenosis [18]. Another interesting case was reported by Silvestri et al. of a patient who had a temporoparietal infarction and was found to have brachiocephalic and subclavian stenosis due to goiter compression [19].

The main cause of our patient’s presentation is yet to be identified. It is important to highlight that the clinical features of hyperthyroidism may be overshadowed by the neurological events. In this case, the thyrotoxic state was identified only due to persistently elevated body temperature, underscoring the importance of a comprehensive evaluation in stroke patients, especially when traditional risk factors are absent.

Treatment of thyrotoxicosis in the setting of stroke presents a challenge. Our patient received antithyroid therapy, beta-blockers, along with gluccocorticooids. Anticoagulation therapy was not initiated. It is to be noted that there is no specific recommendation regarding anticoagulation therapy [20], but it is recommended in case of associated AF [21].

Nonselective beta blockers such as propranolol and esmolol are considered to decrease the sympathetic activity and heart rate in such cases. Both can be received intravenously in emergency cases; however, they are contraindicated in acute systolic heart failure and asthma. Glucocorticoids decrease the conversion of T4 to T3 and prevent adrenal insufficiency. Anti-thyroid medications, such as PTU and methimazole, inhibit thyroid peroxidase enzyme, but PTU is considered superior as it decreases peripheral conversion of T4 to T3. Iodine can be considered as well, by increasing the iodine level and preventing further binding to thyroglobulin. Idoine should be given with a time interval after administering anti-thyroid medications, as their combination may worsen symptoms by increasing thyroid hormone production. Other suggested treatments include cholestyramine, surgical interventions such as thyroidectomy or thyroid ablation and plasmapheresis [22,23].

## Conclusions

This case highlights the potential association between hyperthyroidism and ischemic stroke, underlying the importance of considering hyperthyroidism as a contributing factor in particularly young patients presenting with large-vessel or cryptogenic strokes. It is important to consider other co-existing metabolic abnormalities as well. Early recognition and appropriate management of thyroid dysfunction, alongside standard stroke interventions, are essential to optimize clinical outcomes. Increased awareness of endocrine factors can be helpful for clinicians to evaluate and manage complicated stroke presentations.
